# Association between nonalcoholic fatty liver disease and peripheral neuropathy in US population, a cross-sectional study

**DOI:** 10.1038/s41598-023-32115-4

**Published:** 2023-03-31

**Authors:** Xi Gu, Dou Tang, Yan Xuan, Ying Shen, Lei Qun Lu

**Affiliations:** Department of Endocrinology, RuiJin Hospital Lu Wan Branch, Shanghai Jiaotong University School of Medicine, No. 149 Chongqing South Road, Shanghai, China

**Keywords:** Peripheral neuropathies, Non-alcoholic fatty liver disease

## Abstract

Nonalcoholic fatty liver disease (NAFLD) has become an important risk of type 2 diabetes mellitus (T2DM). Peripheral neuropathy (PN) is regarded as one of the main microvascular complications of diabetes. But the association of NAFLD with PN is still unclear. We aimed to investigate the association between NAFLD and PN in US population by conducting a cross-sectional study. We enrolled 3029 participants aged 40–85 years from National Health and Nutrition Examination Survey (NHANES) 1999–2004. NAFLD was defined as a US Fatty Liver Index (FLI) score ≥ 30, and PN was defined as having one or more insensate areas on either foot. Participants were divided into two groups (with or without PN). We performed multivariate logistic regression models to evaluate the association between NAFLD and PN. Subgroup analyses were used to find out whether the association was stable in different stratified groups. Sensitivity analyses were conducted to assess the robustness of the results. All the analyses were weighted. Among the individuals, 524 (17.3%) had PN and 1250 (41.27%) had NAFLD. In the multivariate logistic regression models, NAFLD was associated with an increased risk of PN (OR 1.44 [1.03 ~ 2.02]) after fully adjusting for covariates. In the subgroup analyses, NAFLD was significantly associated with PN in the age group (40–64 years), compared with those in the age group (65–85 years), (P for interaction: 0.004). The results of association of NAFLD with PN were stable in sensitivity analyses. In this cross-sectional study among US adults aged 40–85 years old, NAFLD was associated with an increased likelihood of prevalent PN.

## Introduction

Nonalcoholic fatty liver disease (NAFLD) has become a very common condition that affected public health. A recent systematic review and meta-analysis^1^ indicated that the pooled global prevalence of NAFLD was likely to close in on a third, at 29.84%. Just second to South America, the prevalence of NAFLD was 35.3% in North America. NAFLD is believed as a multisystem disease. It not only cause liver fibrosis, cirrhosis and hepatocellular carcinoma, but also becomes an important risk of type 2 diabetes mellitus (T2DM), cardiovascular diseases (CVD), and chronic kidney disease^2,3^. Peripheral neuropathy (PN), of which diabetes is the most common cause^4^, is always difficult to treat and can lead to a poor life quality^5^. The prevalence of PN is increasing particularly in older people^6^. PN also increases the risk of ulcers and amputation among diabetic patients^7^. Although NAFLD increases the risk of developing important extra-hepatic complications, the link between NAFLD and PN is still unclear. Limited studies have explored the link, but the results are not consistent^8^. Furthermore the association is not well studied in US population.

Therefore, we aimed to further identify the relationship between NAFLD, determined by US Fatty Liver Index (FLI), and PN in US population by using the database of National Health and Nutrition Examination Survey (NHANES) from 1999 to 2004. We hypothesized that NAFLD was positively associated with PN after adjusting for confounding factors, including Body mass index and diabetes.

## Methods

### Study population

**Figure 1:**
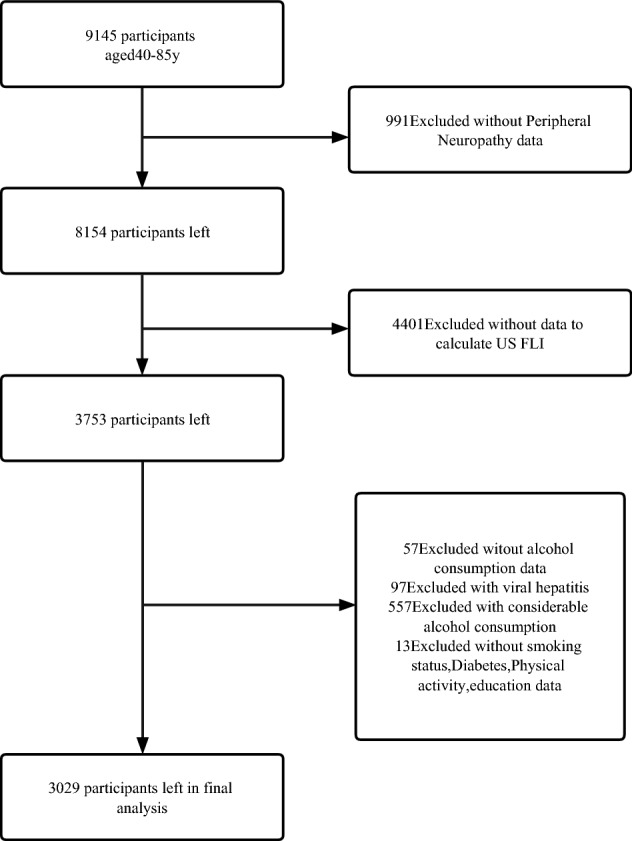
Flow diagram of the screening and enrollment of study participants.

We used data from NHANES to conduct this study. NHANES is an ongoing program of studies in the United States and the data from this survey will be used to help develop sound public health policy, direct and design health programs, and services in this country. The NHANES interview includes demographic, socioeconomic, dietary, and health-related questions. All information of the participants collected in the survey is confidential and privacy is protected by public laws. The NHANES 1999–2004 study protocol (number: Protocol #98-12) was approved by the National Center for Health Statistics (NCHS) Ethics Review Board. All the participants provided written informed consent when enrolling. 9145 participants aged 40–85 years from NHANES 1999–2004 were initially included.991 participants without PN data and 4401 participants without relevant data to calculate US FLI score were excluded. Participants with viral hepatitis, considerable alcohol consumption were also excluded. We further excluded those without alcohol consumption and covariates data, 3029 participants were finally included. More details were shown in Fig. 1. This study was conducted following Strengthening the Reporting of Observational Studies in Epidemiology (STROBE) guidelines.

### Definition of NAFLD

Due to lack of the data of abdominal ultrasonography and liver biopsy, we used US FLI score to determine the diagnosis of NAFLD. USFLI = (e − 0.8073 × non-Hispanic black + 0.3458 × Mexican American + 0.0093 × age + 0.6151 × ln (gamma glutamyl transpeptidase) + 0.0249 × waist circumference + 1.1792 × ln (insulin) + 0.8242 × ln (glucose) − 14.7812)/(1 + e − 0.8073 × non-Hispanic black + 0.3458 × Mexican American + 0.0093 × age + 0.6151 × ln (gamma glutamyl transpeptidase) + 0.0249 × waist circumference + 1.1792 × ln (insulin) + 0.8242 × ln (glucose) − 14.7812) × 100. According to previous studies^9,10^, we defined the participants had NAFLD if the US FLI score was equal 30 or greater. At the same time, we excluded the subjects who had a history of viral hepatitis or had a considerable alcohol consumption (≥ 20 g/day for men and ≥ 10 g/day for women).

### Definition of PN

Health technicians tested participants’ foot sensation by using a standard monofilament (5.07 gauge Semmes–Weinstein nylon) to press at three sites of each foot. If the responders couldn’t correctly answer the question or were not sure where the monofilament was applied, the site would be considered insensate. Based on previous research^11–13^, PN was defined as having at least one or more insensate site on either foot.

### Definition of covariates

Alcohol consumption was obtained from the first day dietary survey data.

Body mass index (BMI) was calculated as weight in kilograms divided by height in meters squared. Poverty income ratio (PIR) was a ratio of family income to poverty threshold, it was divided into three groups: low income (≤ 1.3), medium income (> 1.3 and  ≤ 3.5), and high income (> 3.5). Smoking status was determined as never smokers (smoked less than 100 cigarettes over a lifetime), former smokers (smoked more than 100 cigarettes before but don’t smoke any more) and current smokers (smoked more than 100 cigarettes in a lifetime and smoke someday/everyday now). Physical activity (PA) was determined by whether the individuals participated in walking or bicycling. Participants who met any one of the following conditions were identified as having diabetes: (1) Fasting plasma glucose ≥ 7.0 mmol/l, (2) HbA1c ≥ 6.5%, (3) random plasma glucose ≥ 11.1 mmol/l, (4) ever told by doctor to have diabetes, (5) using any anti-glycemic drugs. The diagnosis of hypertension consists of three components as below: (1) during the physical examination, systolic blood pressure ≥ 140 mmHg or diastolic blood pressure ≥ 90 mmHg was found greater than or equal to 3 times. (2) Ever told by doctor to have hypertension. (3) Taking prescription for hypertension. If anyone of the above conditions was fulfilled, the responder was determined as having hypertension. Hyperlipidemia was diagnosed if the participants met one of the three conditions: (1) total cholesterol ≥ 5.17 mmol/l or low-density lipoprotein (LDL) ≥ 3.36 mmol/l or High-density lipoprotein (HDL) < 1.03 mmol/l (male)/1.29 mmol/l (female), (2) triglycerides ≥ 1.69 mmol/l, (3) taking lipid-lowering drugs.

### Statistical analyses

In accordance with the requirements of NCHS, all the analyses in this study were conducted by using complex sampling design and sample weights. We used the following formula calculate the sample weight: sample weight = 2/3*four-year subsample fasting weight (years in 1999–2000, 2001–2002), or 1/3*two-year subsample fasting weight (years in 2003–2004). Continuous variables were described as mean with standard error (SE) or median (interquartile range [IQR]) and categorical variables were presented by number (unweighted) and percentage (weighted). Comparisons between two groups were performed with the student t test or the Mann–Whitney U test for continuous variables or χ^2^ test for categorical variables. We used univariable and multivariable binary logistic regression model to test the association of NAFLD with PN in four different models. All significant covariates in the univariate analyses and clinical related risk factors were included in the multivariate model. Model 1 was the crude model. In model 2, we adjusted for age, gender, ethnicity. In Model 3, we additionally adjusted for education, smoking status, alcohol consumption, PA, PIR, Hypertension and Hyperlipidemia. In Model 4, we further adjusted for Diabetes, BMI. We also performed a subgroup analyses according to age categories (< 65 or ≥ 65), BMI categories (< 30 or ≥ 30), gender and diabetes status. To assess the robustness of the findings, we performed sensitivity analyses by excluding participant with vitamin B12 deficiency (serum vitamin B12 ≤ 148 pmol/l), and excluding those taking the ‘Phenytoin sodium’, an anticonvulsant medication, the two models were adjusted for age, gender, ethnicity, education, smoking status, alcohol consumption, PA, PIR, Hypertension, Hyperlipidemia, Diabetes and BMI. At last, we calculated the ‘NAFLD liver fat score’ and the values greater than -0.640 were regarded as having NAFLD^14^. Considering the factors of collinearity, this model was adjusted for age, gender, ethnicity, education, smoking status, alcohol consumption, PA, PIR, Hypertension and Hyperlipidemia.

We conducted a Mediation Analysis using the ‘bruceR’ package. Process function (model 4) in this package was performed to identify whether diabetes influence the relationship between NAFLD and PN as the mediator. If the 95% Bootstrap confidence intervals of the estimated indirect effect did not include number zero, the mediation effect were considered statistically significant^15^.

All the results of associations were measured using odds ratios (OR) and 95% confidence intervals (CI). We replaced the missing values of BMI, PIR, and LDL with the mean values. All statistical analyses were performed with the statistical software packages R version 4.2.1 (The R Foundation, Shanghai, China) and Free Statistics software versions 1.7.1. A 2-sided P < 0.05 was considered statistically significant.

## Results

### Baseline characteristics of this study population

In this study, a total of 3029 participants were included in the final analyses. Among them, 1405 were Male (46.38%), and the mean age was 57.5 (SE0.31) years old. In this population, 524 (17.3%) had PN and 1250 (41.27%) had NAFLD which was defined by US FLI. We found that the participants with PN were older (64.56 [0.70]), had higher US FLI score (33.61 [1.36]), had lower alcohol consumption (0.4 [0.1]), more likely be Male, had lower education levels, had a lower family income, more likely be former smokers, had Diabetes and Hypertension than those without PN. Above results were presented in Table 1.

**Table 1:** Baseline characteristics of adult stratified by PN status.

Variable	Total	No PN (n = 2505)	PN (n = 524)	*P* value
US FLI	26.67 (0.69)	25.67 (0.74)	33.61 (1.36)	< 0.0001
Age (years)	57.50 (0.31)	56.48 (0.35)	64.56 (0.80)	< 0.0001
BMI (kg/m^2^)	28.68 (0.19)	28.62 (0.21)	29.11 (0.36)	0.23
Alcohol (g/day)	0.65 (0.08)	0.69 (0.09)	0.40 (0.10)	0.03
Fasting glucose (mmol/l)	5.88 (0.05)	5.83 (0.04)	6.19 (0.14)	0.01
Fasting insulin (pmol/l)	71.73 (1.52)	69.61 (1.67)	86.52 (4.60)	0.002
HbA1c (%)	5.65 (0.03)	5.61 (0.03)	5.91 (0.08)	< 0.001
HDL (mmol/l)	1.35 (0.01)	1.36 (0.01)	1.28 (0.02)	0.002
LDL (mmol/l)	3.27 (0.02)	3.28 (0.03)	3.17 (0.06)	0.07
WBC (1000 cells/μl)	6.78 (0.06)	6.74 (0.06)	7.08 (0.18)	0.07
Alt (u/l)	21.00 (17.00, 27.00)	21.00 (17.00, 27.00)	21.00 (17.00, 27.00)	0.45
Ast (u/l)	22.00 (19.00, 26.00)	22.00 (19.00, 26.00)	22.00 (19.00, 27.00)	0.38
γGT (u/l)	20.00 (15.00, 31.00)	20.00 (15.00, 30.00)	22.00 (16.00, 31.00)	0.02
Fibrosis-4	1.03 (0.78, 1.42)	1.01 (0.77, 1.36)	1.31 (0.89, 1.75)	< 0.0001
HOMA-IR	2.36 (1.51, 3.92)	2.31 (1.47, 3.79)	2.80 (1.82, 4.81)	< 0.0001
CRP (mg/dl)	0.24 (0.11, 0.53)	0.24 (0.10, 0.52)	0.23 (0.13, 0.56)	0.13
TG (mmol/l)	1.45 (1.04, 2.13)	1.43 (1.03, 2.12)	1.54 (1.07, 2.26)	0.2
Gender				
Female	1624 (53.62)	1402 (57.96)	222 (45.66)	< 0.0001
Male	1405 (46.38)	1103 (42.04)	302 (54.34)	
Ethnicity				
Non-Hispanic White	1660 (54.8)	1377 (78.18)	283 (75.85)	0.4
Non-Hispanic Black	488 (16.11)	399 (8.43)	89 (9.86)	
Other Hispanic	114 (3.76)	96 (4.30)	18 (5.81)	
Mexican American	686 (22.65)	561 (4.74)	125 (5.36)	
Other Race	81 (2.67)	72 (4.34)	9 (3.11)	
Education				
Less than High school	1070 (35.33)	841 (20.63)	229 (30.23)	0.01
High school	693 (22.88)	588 (26.29)	105 (22.15)	
College	1266 (41.8)	1076 (53.08)	190 (47.62)	
PIR				
≤ 1.3	714 (23.57)	564 (15.20)	150 (21.75)	0.002
> 1.3–3.5	1384 (45.69)	1121 (40.31)	263 (47.92)	
> 3.5	931 (30.74)	820 (44.49)	111 (30.33)	
PA				
No	2430 (80.22)	2006 (81.17)	424 (78.24)	0.27
Yes	599 (19.78)	499 (18.83)	100 (21.76)	
Smoke				
Never	1495 (49.36)	1249 (49.24)	246 (43.11)	0.02
Former	1066 (35.19)	849 (33.21)	217 (42.66)	
Now	468 (15.45)	407 (17.55)	61 (14.23)	
Diabetes				
No	2417 (79.8)	2057 (86.09)	360 (72.87)	< 0.0001
Yes	612 (20.2)	448 (13.91)	164 (27.13)	
Hypertension				
No	1317 (43.49)	1128 (52.01)	189 (41.69)	0.003
Yes	1711 (56.51)	1376 (47.99)	335 (58.31)	
Hyperlipidemia				
No	496 (16.38)	411 (16.55)	85 (15.25)	0.55
Yes	2533 (83.62)	2094 (83.45)	439 (84.75)	
NAFLD				
No	1779 (58.73)	1517 (66.06)	262 (51.34)	< 0.0001
Yes	1250 (41.27)	988 (33.94)	262 (48.66)	

*PN* peripheral neuropathy, *US FLI* U.S. Fatty Liver Index, *BMI* body mass index, *HbA1C* glycosylated hemoglobin A1C, *HDL* high-density lipoprotein, *LDL* low density lipoprotein, *WBC* white blood cell, *Alt* alanine transaminase, *Ast* aspartate transaminase, *γGT* gamma glutamyl transferase, *HOMA-IR* homeostasis model assessment of insulin resistance, *CRP* C-reactive protein, *TG* triglyceride, *PIR* poverty income ratio, *PA* physical activity, *NAFLD* non-alcoholic fatty liver disease.

### Association between univariate and PN in logistic regression model

We performed the univariant logistic regression analyses to assess the association between univariate and PN. In these regressions, we found that age (OR 1.05), Male (OR 1.64), Former smoker (OR 1.47, compared with never smoker), having Diabetes (OR 2.3), having Hypertension (OR 1.54), having NAFLD (OR 1.85) were positively associated with PN. In addition, compared with education of less than High school, having education of High school (OR 0.58) or college (OR 0.61) was inversely associated with PN. Having more family income (OR 0.48) was also negatively associated with PN. The results were shown in Table 2.

**Table 2:** Association between univariate and PN in logistic regression model.

Variable	OR (95% CI)	*P* value
Age (years)	1.05 (1.04–1.07)	< 0.0001
Gender		
Female	Ref	
Male	1.64 (1.31–2.06)	< 0.0001
Ethnicity		
Non-Hispanic White	Ref	
Mexican American	1.17 (0.84–1.61)	0.350
Non-Hispanic Black	1.21 (0.87–1.67)	0.250
Other Hispanic	1.39 (0.80–2.44)	0.240
Other Race	0.74 (0.32–1.72)	0.470
Education		
Less than High school	Ref	
High school	0.58 (0.39–0.85)	0.01
College	0.61 (0.45–0.84)	0.003
Smoking status		
Never	Ref	
Former	1.47 (1.11–1.94)	0.01
Now	0.93 (0.59–1.46)	0.74
Alcohol consumption (g/day)	0.96 (0.91–1.00)	0.05
Diabetes		
No	Ref	
Yes	2.30 (1.66–3.20)	< 0.0001
Hypertension		
No	Ref	
Yes	1.54 (1.18–1.99)	0.002
Hyperlipidemia		
No	Ref	
Yes	1.10 (0.79–1.53)	0.55
PA		
No	Ref	
Yes	1.20 (0.87–1.66)	0.27
PIR		
≤ 1.3	Ref	
> 1.3–3.5	0.83 (0.61–1.12)	0.22
> 3.5	0.48 (0.32–0.70)	< 0.001
BMI (kg/m^2^)	1.01 (0.99–1.04)	0.23
Fibrosis-4	1.72 (1.41–2.10)	< 0.0001
Advanced fibrosis		
No	Ref	
Yes	3.26 (1.98–5.37)	< 0.0001
NAFLD		
No	Ref	
Yes	1.85 (1.41–2.42)	< 0.0001

*NAFLD* non-alcoholic fatty liver disease, *PN* peripheral neuropathy, *BMI* body mass index, *PIR* poverty income ratio, *PA* physical activity, *OR* odds ratio, *CI* confidence interval, *Ref* reference.

### Association between NAFLD and PN in multivariate logistic regression model

In Model1 (crude model), NAFLD had a higher risk (OR 1.85, 1.41–2.42) of developing PN. Relative to participants without NAFLD, the OR (95% CI) of having PN in those with NAFLD was1.71 (1.28–2.29) in Model 2 (adjusted for age, gender, ethnicity), 1.74 (1.26–2.40) in Model 3 (further adjusted for education, smoking status, alcohol consumption, PA, PIR, Hypertension and Hyperlipidemia) and 1.44 (1.03–2.02) in Model 4 (adjusted for Model 3 plus Diabetes, BMI) respectively. The results could be seen in Table 3.

**Table 3:** Association between NAFLD and PN in multivariate logistic regression model.

Variable	Model 1		Model 2		Model 3		Model 4	
	Crude OR (95% CI)	Crude *P* value	Adj OR (95% CI)	Adj *P* value	Adj OR (95% CI)	Adj *P* value	Adj OR (95% CI)	Adj *P* value
NAFLD								
No	1 (Ref)		1 (Ref)		1 (Ref)		1 (Ref)	
Yes	1.85 (1.41–2.42)	< 0.0001	1.71 (1.28–2.29)	< 0.001	1.74 (1.26–2.40)	0.002	1.44 (1.03–2.02)	0.03

*NAFLD* non-alcoholic fatty liver disease, *PN* peripheral neuropathy, *BMI* body mass index, *PIR* poverty income ratio, *PA* physical activity, *OR* odds ratio, *CI* confidence interval, *Ref* reference.

Model 1: crude model.

Model 2: adjusted for age, gender, ethnicity.

Model 3: adjusted for Model2 plus education, smoking status, alcohol consumption, PA, PIR, Hypertension and Hyperlipidemia.

Model 4: adjusted for Model3 plus Diabetes, BMI.

### Subgroup analyses of association between NAFLD and PN

**Figure 2:**
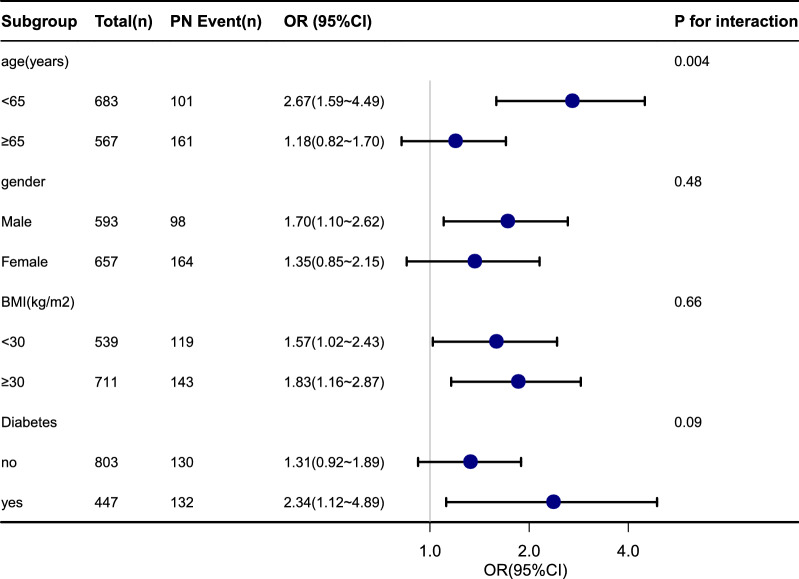
Subgroup analyses of association between NAFLD and PN. *NAFLD* non-alcoholic fatty liver disease, *PN* peripheral neuropathy, *BMI* body mass index, *PIR* poverty income ratio, *PA* physical activity, *OR* odds ratio, *CI* confidence interval, *Ref* reference. Each stratification was adjusted for ethnicity, education, PIR, smoking status, alcohol consumption, PA, Hypertension and Hyperlipidemia.

In the subgroup analyses (Fig. 2), the NAFLD was associated with PN in male participants (OR 1.70, 1.10–2.62) and among subjects who aged 40 to 64 years (OR 2.67, 1.59–4.49) or with Diabetes (OR 2.34, 1.12–4.89). The association was also significantly positive in both BMI groups (< 30 and ≥ 30 kg/m^2^). There was no significant association in participants whose age was 65–85 years and those were female or without Diabetes. The P for interaction was 0.004 in the subgroup of age. The subgroup analyses were adjusted for ethnicity, education, smoking status, alcohol consumption, PA, PIR, Hypertension and Hyperlipidemia.

### Sensitivity analyses

After excluding the participants with vitamin B12 deficiency or taking the anticonvulsant medication ‘Phenytoin sodium’, PN was still associated with NAFLD (OR 1.44, 1.03–2.02). In an additional sensitivity analysis, PN was associated with NAFLD redefined by ‘NAFLD liver fat score’ (OR1.46, 1.09–1.94). The results were presented in Table 4.

**Table 4:** Sensitivity analyses.

Analysis	Tot N	Adj OR (95% CI)	*P* value
Excluding participants with vitamin B12 deficiency^a^	2955		
NAFLD			
No		Ref	
Yes		1.44 (1.03–2.02)	0.03
Excluding participants taking Phenytoin ^a^	3009		
NAFLD			
No		Ref	
Yes		1.44 (1.03–2.02)	0.04
NAFLD defined by NAFLD-liver fat score^b^	3079		
NAFLD			
No		Ref	
Yes		1.46 (1.09–1.94)	0.01

*NAFLD* non-alcoholic fatty liver disease, *BMI* body mass index, *PIR* poverty income ratio, *PA* physical activity, *OR* odds ratio, *CI* confidence interval, *Ref* reference, *Tot N* total number.

^a^Adjusted for age, gender, ethnicity, education, smoking status, alcohol consumption, PA, PIR, Hypertension, Hyperlipidemia, Diabetes and BMI.

^b^Adjusted for age, gender, ethnicity, education, smoking status, alcohol consumption, PA, PIR, Hypertension, Hyperlipidemia.

### Mediation analysis

The mediation analysis indicated that NAFLD could affect PN not only directly but also indirectly through diabetes status after controlling for age, gender, ethnicity, education, smoking status, alcohol consumption, PA, PIR, Hypertension and Hyperlipidemia. The direct and indirect effect (95% bootstrap CI) was 0.037 (0.009–0.066), 0.017 (0.008–0.027) respectively (see Supplementary Table S1 online).

## Discussion

In our study, we identified the positive association between NAFLD and PN in US population after adjustment for related major confounding variate. In the subgroup analyses, the relationship was significant in middle-age group (aged 40–64 years). No interactions were detected in the other subgroups.

To our knowledge, there were not enough similar studies done before. Most of these similar studies have been conducted only in diabetic populations and the results of them were inconsistent. A matched case–control study was conducted in Iran in 2019^16^. They enrolled 935 patients with type 2 diabetes and found that NAFLD was not associated with diabetic peripheral neuropathy (DPN). But in the subgroup, NAFLD with elevated serum liver enzymes was inversely correlated with DPN in the fully adjusted model. In contrast, our study showed that NAFLD was associated with PN in general people. Even after full adjustment for variables, this association remained positive. Similarly, a 2013 study^17^ in Qingdao, China also reported that NAFLD was negatively correlated with the DPN. However, this study did not adjust for covariates, so the results may not be reliable. Another study^18^ in Tianjin, China in 2016 focused on the association of the temporal sequence of NAFLD and type 2 diabetes with diabetic complications. A total of 212 individuals were included. In this study, the patients with NAFLD longer than type 2 diabetes had a lower prevalence of DPN. But no regression model was performed, the results should be regarded prudently. Compared to these two studies in Chinese patients, covariates were fully adjusted in our study and univariate and multivariate logistic regressions were also performed. We noticed a study in Korea in 2014^19^, it concluded that NAFLD was not associated with diabetic neuropathy but still with lower OR of diabetic retinopathy and nephropathy after adjustment for confounding factors. We found the positive association between NAFLD and PN in our study and maybe it is needed to explore whether NAFLD is associated with other microvascular complications in future studies. Another study^20^ recruited 264 individuals with type 2 diabetes from Seoul, Korea. The study suggested that the NAFLD fibrosis score (NFS) and Fibrosis-4 (FIB-4) index were associated with DPN among patients with a high NAFLD liver fat score (> − 0.640). Unlike this study, we used US FLI to define NAFLD and had a similar result. Another two similar studies^21,22^ were conducted previously. One was in Australia and the other was in Italian. They also found NFS was associated with large-fiber peripheral nerve dysfunction or chronic vascular complications among patients with type 2 diabetes. Like these studies, we also evaluated the association of liver fibrosis with PN using FIB-4 score in a univariate regression model. FIB-4 score > 2.67 was defined as advanced fibrosis. FIB-4 and advanced fibrosis were positively associated with PN (Table 2). We also noted another study^23^ that investigated this association in other populations in 2017. The study concluded that NAFLD, diagnosed by ultrasonography, was strongly associated with an increased risk of distal symmetric polyneuropathy in type 1 diabetic adults, independently of several cardio-metabolic risk factors. Similarly, we also found this association remained stable after adjustment for cardio-metabolic risk factors, such as hypertension, hyperlipidemia, and BMI etc. In summary, all these previous studies were conducted in diabetes patients. But in our study, we found the diabetes was a mediator. Moreover, the results of subgroup analyses showed that the association of NAFLD with PN was positive in both group of participants having diabetes or not, the p for interaction did not show the statistical difference.

Currently, the potential mechanisms linking NAFLD with PN are not well known through available data. Some evidence shows that NAFLD may increase the risk of chronic vascular complications through a kind of different pathogenic mechanisms^24^. Intrahepatic fat accumulation is associated with the development of NAFLD and further lead to hepatic and peripheral insulin resistance^2,25^. The insulin resistance in NAFLD is associated with the hypertension, diabetes and dyslipidemia^26,27^. The levels of triglycerides and small, dense LDL cholesterol increase but HDL cholesterol levels decrease in the circulation, which will cause atherosclerosis^28,29^. In addition, the upregulation of multiple pro-inflammatory pathways^30,31^, low levels of adiponectin^31,32^, increased endoplasmic reticulum stress^33^, mitochondrial dysfunction^34,35^ and the increased circulating levels of fibrinogen, factor VIII, coagulation factor and plasminogen activator inhibitor1 (PAI1)^36,37^ are the potential mechanisms to increase the risk of chronic vascular complications in individuals with NAFLD.

### Strength

First, the previous studies were conducted mainly in Asia and Europe. Unlike these studies, our study selected the samples from NHANES in US so that we can evaluate the association in different population from the world. Second, we included more subjects than those in the other similar studies. At the same time, we used the complex design and sample weights to analysis the data according to NHANES requirement. So, the results could represent the general population in US, to a certain extent. Third, we analyzed the data from the general people rather than only from diabetic patients. The results of the subgroup analysis showed that the association of NAFLD with PN may not just limit in the people having diabetes.

### Limitation

First, due to the cross-sectional study design, we could not obtain the definite causal relationship between NAFLD and PN. Second, the age of participants is from 40 to 85 years, this may lead to a selection bias. But as we know, NHANES staff did the examination of the feet sensation in participants who aged 40 years or older. We couldn’t get to know whether this association exists in those aged below 40 years in this database. Last, we determined the define of NAFLD by using US FLI score rather than abdominal ultrasound examination or liver biopsy. Maybe we did not determine the NAFLD more accurately, but there was no ultrasound data in NHANES 1999–2004. Moreover, US FLI is a suitable formula to help diagnosing the NAFLD in general population with an area under the receiver operating characteristic curve (AUROC) of 0.80 (sensitivity, 62%; specificity, 88%)^9^.

### Conclusion

In this cross-sectional study, we identified the statistically significant association between NAFLD and increased risk of PN in general US population. In subgroup analyses, we found the association of NAFLD with PN was significant in the age group (40–64 years).

## Supplementary Information

Supplementary Information.

## Data Availability

All the datasets are available on the NHANES website (http://www.cdc.gov/nchs/nhanes.htm).
