# Supplementary material for: Association between nonalcoholic fatty liver disease and peripheral neuropathy in US population, a cross-sectional study
Source: Sci Rep. 2023 Mar 31;13:5304. doi: 10.1038/s41598-023-32115-4 (PMC10066263; doi:10.1038/s41598-023-32115-4)
Supplement: Supplementary file 1 — Supplementary Information. [file 41598_2023_32115_MOESM1_ESM.docx]

| Table S1 Mediation analysis ^a^ | | | | |
| --- | --- | --- | --- | --- |
|  | Effect | S.E. | *P* | Boot 95%CI |
| Indirect | 0.017 | 0.005 | <0.001 | 0.008~0.027 |
| Direct | 0.037 | 0.015 | 0.011 | 0.009~0.066 |
| Total | 0.054 | 0.014 | <0.001 | 0.026~0.082 |

(SE and CI are estimated based on 5000 Bootstrap samples.)

a: controlling for age, gender, ethnicity, education, smoking status,

alcohol consumption, PA, PIR, Hypertension and Hyperlipidemia.

S.E.: standard error; Boot: Bootstrap; CI: Confidence Interval.

PIR: poverty income ratio; PA: physical activity.
